# Evaluation of the Efficacy of Treatment for Convergence Insufficiency with a New Digital Mobile Platform: A Comparative Preliminary Study

**DOI:** 10.3390/vision10020031

**Published:** 2026-05-17

**Authors:** Alba Pina-Balofer, David P. Piñero, Miranda Buigues, Carlo Cavaliere-Ballesta, Sergio Viudes, Laurent Bataille

**Affiliations:** 1 Visitrain S.L., Science Park, University of Alicante, 03005 Alicante, Spain; apina@visitrain.onmicrosoft.com (A.P.-B.); ccavaliere@visitrain.onmicrosoft.com (C.C.-B.); lbataille@visitraintherapies.com (L.B.); 2 Department of Optics, Pharmacology and Anatomy, University of Alicante, 03690 San Vicente del Raspeig, Alicante, Spain; mirandabuigues@icloud.com; 3 Department of Ophthalmology, Vithas Medimar International Hospital, 03016 Alicante, Spain; 4 Baviux Apps and Game Studio, 03203 Elche, Alicante, Spain; sviudes@baviux.com; 5 Department of Computational Science and Artificial Intelligence, University of Alicante, 03690 San Vicente del Raspeig, Alicante, Spain

**Keywords:** convergence insufficiency, vision therapy, digital platforms, vergence, Visitrain VG

## Abstract

The objective of this study was to evaluate the efficacy of a novel digital platform (Visitrain VG, Alicante, Spain) as a visual rehabilitation tool for patients with convergence insufficiency (CI), in comparison with conventional in-office vision therapy (VT) supplemented with home reinforcement exercises. A retrospective comparative study was conducted comprising 33 patients diagnosed with CI, allocated into two groups: a digital group (DG; *n* = 16) receiving treatment with the aforementioned digital platform and a conventional group (CG; *n* = 17) undergoing conventional vision therapy. Binocular vision clinical parameters were assessed at baseline, one month, and three months of follow-up, including near point of convergence (NPC), positive fusional vergence (PFV), and binocular accommodative facility (BAF). Both groups demonstrated significant improvements following three months (*p* < 0.050). At the one-month evaluation, the CG showed a more rapid clinical response, with statistically significant between-group differences being observed in the NPC (*p* = 0.004) and near PFV (*p* = 0.040) compared with the DG. Nevertheless, at the three-month follow-up, no significant differences were found between the groups (*p* ≥ 0.060). The digital platform under investigation appears to constitute an effective therapeutic alternative to conventional vision therapy, albeit with a comparatively slower initial clinical response rate. It may be particularly indicated for patients requiring greater scheduling flexibility or those with limited access to in-office clinical care. Prospective controlled clinical trials are warranted to corroborate these preliminary outcomes.

## 1. Introduction

Convergence insufficiency (CI) is one of the most prevalent disorders of binocular vision. Its prevalence varies widely depending on the population under study, ranging from 2.25% to 33%, with an estimated prevalence of approximately 6.8% in the pediatric population when clinically significant cases are considered [1,2]. CI is characterized by the inability to achieve or sustain accurate convergence during near vision tasks [3]. This condition is associated with a range of visual symptoms, including blurred vision, diplopia during prolonged near work, headaches related to visual effort, and accommodative difficulties when alternating between near and distance vision [4].

The diagnosis of CI is based on the evaluation of specific clinical signs and symptoms. Based on the normative cutoff values defined by different authors and revised in the systematic review by Cacho et al., the diagnostic criteria can include: a difference between distance and near exophoria ≥ 4 Δ, with greater exophoria at near; a near point of convergence (NPC) break point ≥ 6 cm; and a near positive fusional vergence (PFV) break point ≤ 15 Δ [5]. Furthermore, symptom assessment using validated questionnaires, such as the Convergence Insufficiency Symptom Survey (CISS), constitutes a valuable diagnostic tool, whereby a score ≥ 16 is considered indicative of clinically significant symptomatology [5,6].

According to its etiology, CI is primarily considered a functional disorder arising from alterations in the interaction between the accommodative and vergence systems [7]. Vision therapy (VT) represents the most widely supported treatment modality for CI. Two main therapeutic approaches have been described: office-based vision therapy with home reinforcement (OBVT) and home-based vision therapy (HBVT). OBVT has been demonstrated to be the most efficacious option, with reported success rates of up to 87.5% and clinically significant improvements observed after approximately 8 weeks of treatment. In contrast, HBVT exhibits comparatively lower efficacy, although it may prove beneficial in mild cases or as maintenance intervention. VT protocols are typically implemented over several weeks and comprise supervised in-office sessions combined in conjunction with structured home exercise programs [8].

In recent years, digital systems applied to vision therapy have emerged as innovative technological tools for the assessment and management of CI. These systems encompass computer-assisted visual training programs, virtual reality devices, eye-tracking systems, and telerehabilitation platforms. Such tools enable the analysis of ocular motor responses and the delivery of controlled visual stimuli, the real-time monitoring of vergence responses, and progressive adaptation of exercise difficulty in accordance with patient advancement. Furthermore, their interactive and immersive nature may enhance treatment adherence, particularly in individuals with limited availability for in-person sessions, and may contribute to optimizing therapeutic outcomes [9,10,11,12]. Nevertheless, despite the growing implementation of digital vision therapy systems, the available scientific evidence remains limited with respect to their therapeutic efficacy, optimal treatment duration, and clinical outcomes relative to conventional therapeutic approaches [10].

Therefore, the objective of the present study was to evaluate the efficacy of the digital platform Visitrain VG (Visitrain S.L., Alicante, Spain) in the treatment of CI, in comparison with conventional vision therapy, through the analysis of changes in key clinical parameters of binocular vision.

## 2. Materials and Methods

### 2.1. Patient Selection

A retrospective, non-randomized comparative study was conducted comprising two groups, in which a total of 33 patients aged between 5 and 46 years were evaluated at the Advanced Clinical Optometry Unit of the Department of Ophthalmology at Hospital Vithas Medimar Internacional (Alicante, Spain). This study was conducted in accordance with the principles of the Declaration of Helsinki and received ethical approval from the Ethics Committee of the University of Alicante (Code 2025 022). Patients were allocated into two groups:

- Digital group (DG): It comprised 16 patients aged between 6 and 46 years who underwent treatment using the Visitrain VG platform in a home-based vision therapy (HBVT) program over a period of 3 months.
- Conventional group (CG): It comprised 17 patients aged between 5 and 45 years who followed a conventional vision therapy protocol combining supervised in-office sessions with structured home exercises (OBVT) over a period of 3 months.

For both groups, inclusion criteria were established based on the diagnostic criteria of CI, using as reference the normative cutoff values described in the systematic review by Cacho et al., which represents a benchmark reference for the diagnosis for this condition, as outlined in the Section 1 [5]. Additionally, only patients who completed the full vision therapy program and provided informed consent were eligible for inclusion. Exclusion criteria comprised: patients unable to adequately comprehend or perform the required clinical tests during the initial assessment; cases of CI associated with acquired brain injury or neurological conditions; and patients who did not complete the prescribed vision therapy program.

### 2.2. Examination Protocol

This study was organized into three visits. At the baseline visit, each patient underwent a full clinical evaluation, and eligibility according to the inclusion and exclusion criteria was verified. Subsequently, two follow-up visits were conducted: one 30 days following the initiation of vision therapy and another at 3 months. Following the initial assessment, an individualized training program was planned for each group in accordance with the protocols described below. The follow-up visits at 30 days and at 3 months included the same tests as the baseline evaluation, with the exception of stereopsis measurement and monocular accommodative facility assessment.

All patients underwent a comprehensive visual examination at baseline prior to commencing vision therapy. This comprised the measurement of uncorrected and corrected visual acuity at distance (5 m) and near (40 cm), assessment of refractive status, the Worth four-dot test at distance and near, and measurement of heterophoria by means of the cover test at near (40 cm) and distance. The NPC was measured using an accommodative stimulus, recording break and recovery points. The break and recovery points of negative (NFV) and positive fusional vergence (PFV) were assessed at both near and distance using a prism bar. Monocular (MAF) and binocular accommodative facility (BAF) were additionally evaluated, along with stereopsis using the TNO stereoscopic test.

The Convergence Insufficiency Symptom Survey (CISS) questionnaire was administered exclusively in the DG, as the clinical records corresponding to the CG were retrospectively retrieved from previous years, during which no standardized protocol had been established for the systematic assessment of symptomatology using validated questionnaires.

#### 2.2.1. Conventional Visual Therapy Protocol

**Figure 1:**
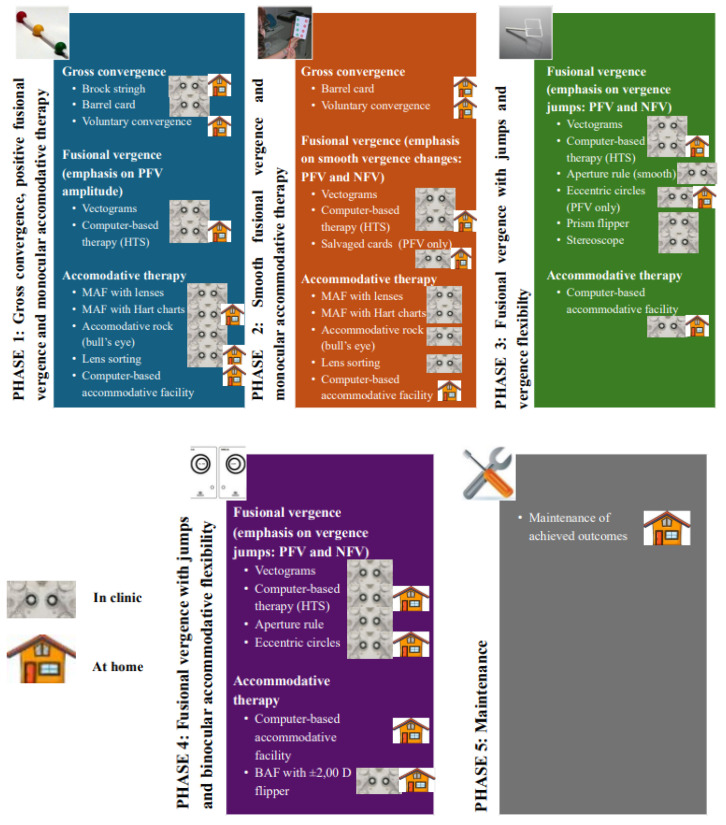
CITT vision therapy protocol used in many clinical trials on the efficacy of vision therapy for convergence insufficiency.

In the CG, the therapeutic protocol employed was that established by the Convergence Insufficiency Treatment Trial (CITT) study group [6,13]. Figure 1 illustrates the different phases of the visual rehabilitation program. In-office sessions ranged from 45 to 60 min in duration, while home-based sessions lasted approximately 15 min, with a prescribed frequency of five sessions per week. The total duration of vision therapy program was 12 weeks [6,13].

The vision therapy program was structured into progressive phases designed to train distinct aspects of visual function, administered both in-office and at home according to the nature of each visual exercise (Figure 1):

- Phase 1: Training of gross convergence, positive fusional vergence, and monocular accommodative training.
- Phase 2: Training of smooth fusional vergence and monocular accommodative training.
- Phase 3: Training of jump fusional vergence and vergence facility.
- Phase 4: Training of jump fusional vergence and binocular accommodative facility.
- Phase 5: Maintenance of results achieved through home-based exercises.

#### 2.2.2. Digital Visual Therapy Protocol

In the DG, daily digital visual rehabilitation sessions of 15 min were prescribed, with a minimum frequency of five sessions per week. Participants installed and operated the Visitrain VG app on their own personal mobile devices. Treatment adherence was remotely monitored by the supervising optometrist through the session data recorded and stored by the platform.

### 2.3. Visual Therapy Software: Visitrain VG

Visitrain VG is a personalized vision therapy platform developed by technology-based company Visitrain S.L., located at the Science Park of the University of Alicante. In the present study, it was employed as a therapeutic training tool for patients with CI, with the aim of improving vergence, sensory, and oculomotor skills, thereby reducing associated symptoms and restoring the visual functions involved in binocular vision control [14].

**Figure 2:**
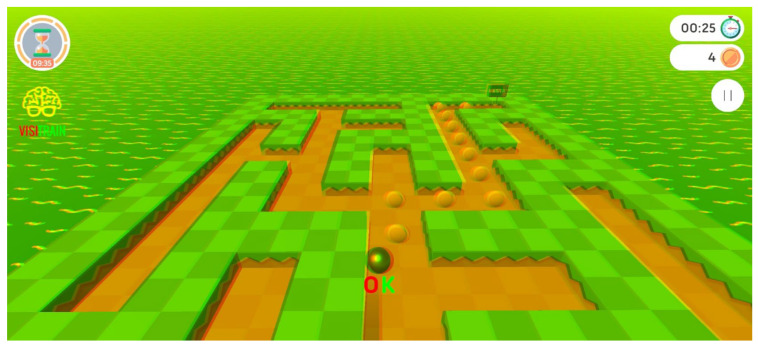
Screenshot of the Visitrain VG mobile video game (Visitrain S.L.).

Training was conducted using red–green anaglyphic glasses for dichoptic stimulus presentation, enabling the dissociation of visual stimuli between each eye independently. This approach also facilitated the detection and control monocular suppression during digital vision therapy sessions. Saccadic eye movements were trained through maze-like environments, in which the displacement of the visual stimulus was synchronized with the rotation or physical movement of the mobile device on which the exercise was performed (Figure 2) [14].

The platform incorporates an adaptive difficulty adjustment system that progressively modifies training parameters in accordance with the patient’s performance, based on the PEST (Parameter Estimation by Sequential Testing) methodology. Visual stimuli were designed to generate convergence demand, thereby promoting progressive improvement in fusional vergence amplitude. The supervising optometrist was able to remotely monitor patient progress and individually adjust training parameters, with the automatic recording of the date and duration of each session [14].

During training, patients were required to track moving visual targets while maintaining appropriate focus as a function on the distance and direction of the presented stimuli [14].

### 2.4. Statistical Analysis

The data were analyzed using SPSS software, version 24.0 (IBM, Armonk, NY, USA). For all quantitative variables, descriptive statistics were calculated, including means, medians, standard deviations, and ranges. The normality of the distributions was assessed using the Shapiro–Wilk test. To assess the statistical significance of differences between the groups at each visit, Student’s *t*-test for independent samples was applied when data were normally distributed, whereas the Mann–Whitney U test was applied when data were non-normally distributed. A *p*-value < 0.050 was considered statistically significant. *p*-Values were adjusted with the Benjamini–Hochberg procedure to control the risk of type I error due to the multiple comparisons done.

## 3. Results

### 3.1. Baseline Characteristics

Various variables related to visual function and visual perceptual performance were compared before and after therapy, assessing differences between the groups using means, medians, and *p*-values to determine statistical significance. Table 1 compares the pre-therapy results of the two patient groups: DG and CG.

A total of 33 patients were allocated in two distinct groups: the DG comprised 16 patients with a mean age of 18.6 years (range, 6.0 to 46.0 years), and the CG comprised 17 patients with a mean age of 13.1 years (range, 5.0 to 45.0 years). No significant difference in age was observed between the groups (*p* = 0.180), suggesting that both groups were comparable in this demographic characteristic. Regarding uncorrected distance visual acuity (UDVA), no significant differences were found between the groups in either the right eye (RE) and left eye (LE) (*p* = 0.620 and *p* = 0.680, respectively). Similarly, corrected distance visual acuity (CDVA) showed no significant differences between the groups (RE: *p* = 0.680; LE: *p* = 0.280), indicating that baseline visual acuity was comparable between the two groups.

The measurement of heterophoria using the cover test at near revealed a difference that, although not statistically significant (*p* = 0.070), suggested a trend toward less near deviation in the CG. Regarding the NPC, no significant differences were found between the groups in either break or recovery values (*p* = 0.850 and *p* = 0.290, respectively). However, for variables related to fusional vergence, slightly higher baseline values were observed in the DG. For near NFV, both break and recovery showed a trend toward higher values in the DG (*p* = 0.070 in both cases), although statistical significance was not reached. More notably, for near PFV, a significant difference between the groups was found exclusively for the recovery measure (*p* = 0.040).

Measures of monocular accommodative facility (MAF RE and MAF LE) and BAF showed no significant differences between the groups (*p* = 0.940, 0.760, and 0.490, respectively), indicating similar baseline accommodative facility in both groups. Finally, regarding stereopsis, although mean values tended to be better in the DG (120.00 vs. 82.70 s of arc), this difference did not reach statistical significance (*p* = 0.290).

### 3.2. One-Month Outcomes

**Figure 3:**
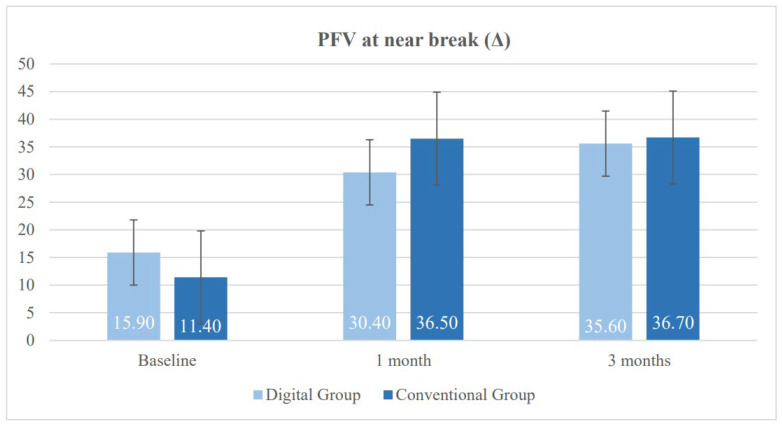
Changes in near positive fusional vergence (PFV) break in the digital group and the conventional group during follow-up.

**Figure 4:**
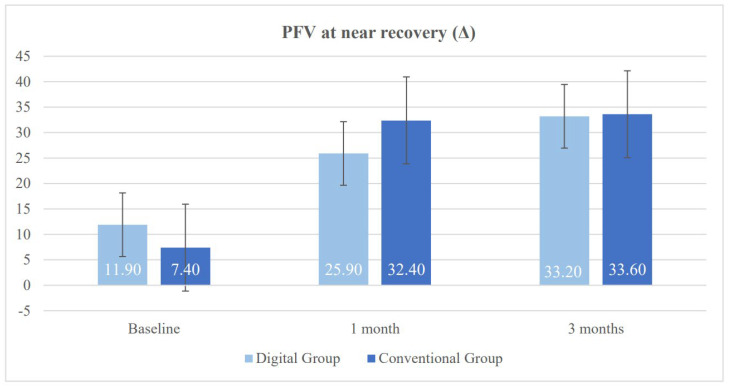
Changes in near positive fusional vergence (PFV) recovery in the digital group and the conventional group during follow-up.

Table 2 presents the results of both study groups after one month of treatment. Significant differences were found in several of the evaluated variables between the DG and the CG. First, a significant difference was observed in heterophoria measured using the cover test at near, with a greater tendency toward near exodeviation in the DG (*p* = 0.020). This trend had already been observed prior to the initiation of vision therapy and persisted thereafter. Regarding the NPC (normal value < 6 cm), both groups showed improvement; however, the degree of improvement was smaller in the DG. Consequently, significant between-group differences were found in both NPC break and recovery values (*p* = 0.004). With respect to fusional vergence, the CG achieved higher values in PFV break, which was the only parameter showing a statistically significant difference in favor of the CG (*p* = 0.040) (Figure 3 and Figure 4). The CG also obtained better results in binocular accommodative facility, exceeding the normal threshold of 6 cycles per minute on average, although this difference did not reach statistical significance (*p* = 0.080).

### 3.3. Three-Month Outcomes

**Figure 5:**
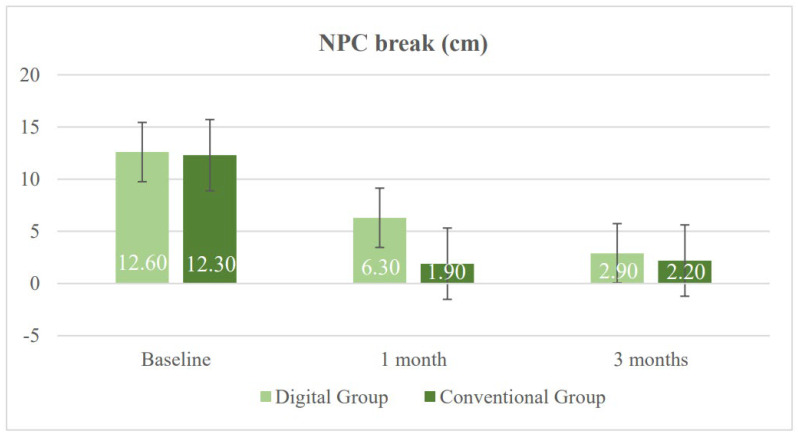
Changes in near point of convergence (NPC) break in the digital group and the conventional group during follow-up.

**Figure 6:**
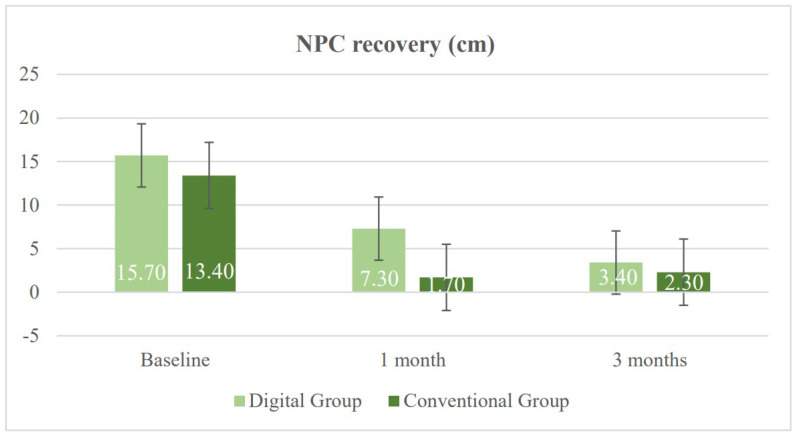
Changes in near point of convergence (NPC) recovery in the digital group and the conventional group during follow-up.

Three-month outcomes were analyzed to assess whether the previously observed functional visual improvements had been sustained over time, as shown in Table 3. At this follow-up point, the findings suggest stabilization of the observed changes, with fewer statistically significant differences between the groups, and no clinically relevant differences remaining. Regarding heterophoria measured using the cover test at near, a persistent tendency toward greater exophoria was observed in the DG, with the between-group difference remaining at the threshold of statistical significance (*p* = 0.050). Regarding the NPC, no significant differences were found between the groups in either break or recovery values (*p* = 0.500 and *p* = 0.470, respectively) (Figure 5 and Figure 6).

Near negative fusional vergence showed a favorable trend in the DG, with higher mean values in both break and recovery points; however, the differences did not reach statistical significance (*p* = 0.110 for break and *p* = 0.060 for recovery). Regarding near PFV, neither break nor recovery showed significant differences between the groups at this final follow-up point (*p* > 0.700) (Figure 3 and Figure 4).

Finally, regarding BAF, no significant differences between the groups nor substantial changes over time were observed (*p* = 0.690). Both groups showed similar values at 30 days and at three months, indicating that the ability to rapidly alternate focus between distances remained stable throughout the study.

Figure 3 illustrates the results of near PFV break, measured in prism diopters (Δ), in both study groups. A significant improvement in near PFV break was observed in both groups following treatment, indicating enhanced convergence ability. The DG demonstrated sustained progression, while the CG achieved greater improvements within the first month, stabilizing by the third month. At the end of follow up, differences between the groups were minimal, demonstrating that both therapeutic approaches were effective.

Figure 4 illustrates the results of near PFV recovery, measured in prism diopters (Δ), for both groups. A significant improvement was observed in both groups following treatment. The DG showed a progressive and sustained increase, whereas the CG demonstrated a faster gain at one month. By the third month, both groups reached comparable values of approximately 33.00 Δ, suggesting similar medium-term efficacy.

Figure 5 illustrates the results of NPC break, measured in centimeters (cm), in both study groups. A faster reduction was observed in the CG during the first month, while the DG showed a more gradual improvement. By three months, both groups reached values within the normal range, indicating effective recovery. Figure 6 illustrates the results of NPC recovery, measured in centimeters (cm), for both study groups. It reflects a gradual improvement in the DG compared with a sharper initial reduction followed by stabilization in the CG.

**Figure 7:**
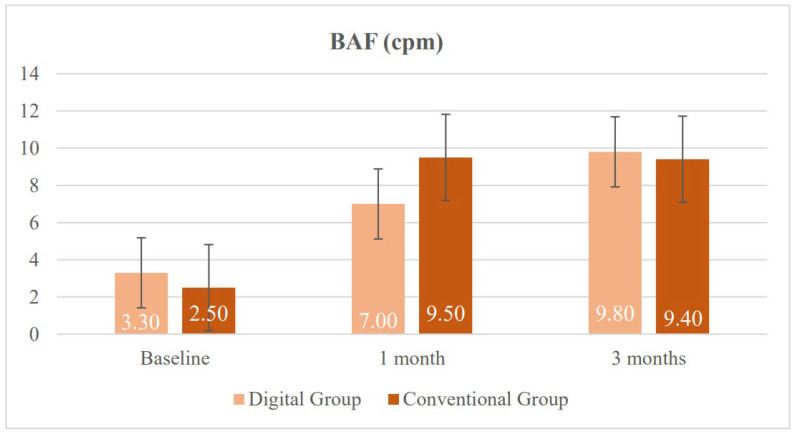
Changes in binocular accommodative facility (BAF) in the digital group and the conventional group during follow-up.

Figure 7 illustrates the positive evolution of BAF, measured in cycles per minute (cpm), in both groups following treatment. At baseline, both groups presented low values (DG: 3.30 cpm; CG: 2.50 cpm), indicating reduced binocular accommodative ability. At one month, a marked improvement was observed in the CG, reaching a mean of 9.50 cpm, exceeding the DG, which improved to a mean 7.00 cpm. By the third month, both groups converged, with the DG reaching a mean of 9.80 cpm and the CG stabilizing at a mean of 9.40 cpm, suggesting that although progress in the DG was more gradual, it ultimately reached comparable levels. Error bars reflected a decrease in variability over time.

Finally, changes in symptomatology evaluated by means of the CISS questionnaire were evaluated but only in the DG. CISS score improved significantly from a mean basal value of 26.90 ± 13.00 to a mean value of 12.00 ± 9.50 (*p* < 0.001) and 7.70 ± 3.40 (*p* < 0.001) after 1 and 3 months of training, respectively. In the CG, compliance with in-office visits was 100%; however, adherence to home-based exercises could not be monitored, as manual exercises were not linked to a digital tracking system. In the DG, the mean compliance was 95.0 ± 10.0%.

## 4. Discussion

The results obtained in this study demonstrate that both conventional vision therapy and the Visitrain VG digital platform can be effective for the treatment of CI, showing significant improvements in key clinical parameters such as NPC, PFV, and BAF. These findings are consistent with previous scientific evidence supporting the effectiveness of vision therapy in the treatment of this condition [1,8,15]. However, relevant differences were observed between the two therapeutic options regarding the temporal evolution of the response. During the first month of treatment, conventional vision therapy showed a faster progression, especially in the NPC and PFV, which is consistent with findings reported in the literature on the effectiveness of supervised in-office supervised therapies [6,16]. Nevertheless, DG showed a more gradual but steady progression, reaching clinically equivalent values to those of the CG by three months. This evolution suggests that although digital therapy may require more time to induce significant clinical changes, its medium-term effectiveness is comparable. Future studies should investigate whether increasing the daily training duration beyond 15 min could lead to more accelerated recovery. It should be also considered that the conventional therapy protocol combined in-office and home sessions on a weekly basis, resulting in greater total time dedicated to visual training per week. This factor may also account for the differential progression observed between the two therapeutic approaches. Treatment adherence may represent another factor contributing to this difference. The records obtained showed no clinically significant differences between the groups in terms of compliance; however, home-based sessions in the CG could not be remotely monitored, as the exercises were not digitalized.

Several studies have previously evaluated the efficacy and safety of digital vision therapy systems for CI [9,10,12,17,18,19,20]. Huston and Hoover [17] assessed the efficacy of the HTS visual therapy program in symptomatic CI in 186 children (5–17 years old). The training consisted of 9–15 min daily exercises, five times per week for six weeks. Following this program, these authors reported improvements in NPC and near PFV, with symptom resolution in 92% of cases, while other treatments were required in 6% of the subjects. Serna et al. [12] reported retrospective results with HTS software in 42 patients (35 of whom received a combination of HTS and push-up exercises), obtaining a significant reduction in the NPC, with 92.8% of subjects achieving an NPC ≤ 6 cm, as well as an improvement in near VFP in 92.8% of subjects and symptom resolution in 64.2% of cases. In that study, the mean duration of therapy was 12.6 weeks. Cooper and Feldman [18] evaluated the effect of the HTS system in subjects with various non-strabismic accommodative and/or binocular anomalies (including CI), and reported a change in a subjective symptom scale from 32.8 ± 8.1 to 20.6 ± 11.5 following therapy, along with changes in convergence amplitude from 22 to 53 Δ and in divergence amplitude from 15 to 25 Δ. More than 75% of the patients completed the program in 40 sessions (equivalent to 8 weeks). On the other hand, Dusek et al. [19] conducted a comparative study in 134 subjects with CI and reading difficulties (ages 7–14), where 51 subjects received 8 Δ base-in prism glasses, 51 subjects received home visual therapy with the HTS system, and 32 subjects received no treatment. The digital therapy proved effective in improving various parameters, including reading speed, and was limited to a maximum of 15 min per day, up to five times per week. In the present study, mean changes at the end of the follow-up (3 months) with the digital system evaluated were −10.2 cm, −7.4 cm, 19.6 Δ, 21.3 Δ, and 6.5 cpm in NPC break, NPC recovery, near PFV break, near PFV recovery and BAF, respectively. These improvements were consistent with and in several cases exceeded those reported in the aforementioned studies. Furthermore, symptomatology was assessed using the validated CISS questionnaire, yielding a mean change of 19.2 points with digital therapy.

The effectiveness observed with the evaluated digital platform may be explained by several factors that distinguish it from other digital visual training systems. Unlike other home-based vision therapy programs, Visitrain VG incorporates gamification elements, automatic difficulty adjustment, interactive visual environments, and remote clinician monitoring. These features promote greater treatment adherence and enable the visual trainer to dynamically adjust the program based on performance and therapeutic compliance. According to Piñero [14], many digital systems have shown limited results due to their less engaging design, lack of supervision, and insufficient personalization, which reduces their effectiveness in medium- to long-term treatments [14]. The Pediatric Eye Disease Investigator Group (PEDIG) [20] conducted a randomized controlled clinical trial in 2016 in 204 children with CI (9–17 years old), comparing three groups: one trained with a customized version of HTS software (75 subjects), one performing near push-up exercises (85 subjects), and one using placebo software (44 subjects). In all groups, therapy was administered five days per week for 12 weeks. At the end of treatment, 23%, 22%, and 16% of the participants in each group, respectively, were considered to have a satisfactory result. These results indicate limited overall efficacy; however, it should be noted that the study was affected by limitations in recruitment and, most notably, in adherence to therapy. It is also worth noting that the HTS system lacks gamification, which may further limit adherence, given that sustained attention and motivation are essential to achieving consistently satisfactory outcomes. These findings suggest that home-based digital therapies cannot function adequately without professional follow-up and monitoring and may need to be combined with in-office sessions or supplementary exercises. In this regard, the American Academy of Ophthalmology established a recommendation in 2021 to combine in-office therapy with home-based exercises for the satisfactory management of CI [21]. More recently, Li et al. [10] reported the results of a comparison between in-office vision therapy following standardized protocols and home-based vision therapy using a custom-developed, non-immersive virtual reality system. No significant differences were found between the groups in binocular and accommodative parameters, with the exception of accommodative flexibility, suggesting the potential of this partly gamified system as a novel vision therapy tool.

The findings of the present study are also consistent with results previously reported by Piñero, who observed that following three months of Visitrain VG therapy, the NPC decreased from 10.8 cm to 3.1 cm, near PFV break increased from 11.2 Δ to 36 Δ, and CISS score decreased significantly from 28.5 to 14.6 points [14]. Significant improvement in saccadic coordination was also reported using eye-tracking systems [14]. Collectively, these findings highlight the potential of well-designed and validated digital systems to replace or complement traditional vision therapy, particularly in patients requiring greater flexibility, remote accessibility, or who have difficulty attending regular in-office sessions.

Despite the positive results, several areas for improvement could further optimize the effectiveness of digital therapy with the evaluated platform. First, increasing session frequency or duration may be beneficial. In the present study, digital sessions lasted 15 min, five times per week. Evidence from the literature suggests that extending sessions to 20 min daily or increasing frequency to six days per week could accelerate clinical improvement and strengthen outcome [11]. Likewise, extending the total treatment duration beyond the three months evaluated could benefit patients with more severe presentations or slower progression. Furthermore, combining digital vision therapy with occasional in-office sessions could help reinforce aspects such as accommodative facility or stereopsis, which require immediate feedback and more controlled exercises. Another improvement would be the incorporation of more advanced monitoring tools, such as real-time eye-tracking sensors or fixation and saccadic analysis systems, which would enable the detection of inadequate response patterns and allow for more precise therapy adjustment [22]. Moreover, the development of therapeutically tailored modules based on specific clinical profiles, using artificial intelligence algorithms, could facilitate greater personalization and more efficient use of each session. Finally, the inclusion of subjective outcome measures in future studies—such as quality-of-life impact, academic performance, or perceived improvement—would allow for a more comprehensive assessment of the overall benefit of these interventions.

This study has several limitations that must be acknowledged. The primary limitation is its retrospective and non-randomized design, which introduces a substantial risk of selection bias and uncontrolled confounding; for this reason, the conclusions drawn should be regarded as preliminary findings to be validated in future controlled studies. A further limitation was the small sample size, although this was partially offset by the inclusion of a control group receiving conventional therapy. The three-month follow-up period was sufficient to detect significant clinical improvements; however, a longer observation period would be advisable to assess the durability and stability of the results. Additionally, CISS assessment was performed only in the EG and not in the CG, which constitutes a further limitation arising from the retrospective design, as it prevents direct between-group comparisons for this variable. Despite these limitations, the findings of the present study reinforce the emerging role of digital platforms as effective and valid tools in the rehabilitation of binocular vision disorders, offering an innovative and accessible alternative that, when properly supervised, can achieve efficacy levels comparable to those of conventional treatments.

## 5. Conclusions

The results of this preliminary study suggest that both conventional vision therapy and the use of a novel gamified digital platform can be effective for the treatment of CI, significantly improving key clinical parameters such as NPC, near PFV, and BAF. Differences in the temporal evolution of clinical outcomes were observed depending on the type of intervention. Conventional therapy seems to provide faster improvement during the first month, particularly in NPC and near PFV, whereas digital therapy reaches comparable values by the third month, suggesting a more gradual but equally efficacious progression. Both therapeutic approaches seem to be able to normalize most functional values, although with slightly more variable responses in the EG, likely due to the personalized and autonomous nature of the treatment. Overall, digital therapy with Visitrain VG appears to represent a valid alternative to conventional therapy, with the additional advantage of remote delivery, which broadens access to treatment in non-face-to-face settings. Its implementation may be particularly beneficial in contexts with limitations in time, resources, or continuous professional supervision. Future studies with larger sample sizes and longer follow-up periods will be needed to consolidate these findings and optimize their clinical application.

## Figures and Tables

**Table 1:** Summary of pre-treatment visual variables in the two study groups: digital group and conventional group.

Mean (SD) Median (Range)	Digital Group (*n* = 16)	Conventional Group (*n* = 17)	*p*-Value	Cohen’s d	95% CI
Age (years)	18.6 (12.7) 13.0 (6.0 to 46.0)	13.1 (10.7) 9.0 (5.0 to 45.0)	0.180	0.47	−0.22 to 1.15
LogMAR UDVA RE	0.01 (0.06) 0.00 (−0.08 to 0.15)	0.00 (0.00) 0.00 (0.00 to 0.00)	0.620	0.22	−0.64 to 1.07
LogMAR UDVA LE	1.00 (0.08) 1.00 (0.90 to 1.20)	0.00 (0.00) 0.00 (0.00 to 0.00)	0.680	0.21	−1.09 to 0.67
LogMAR CDVA RE	0.00 (0.04) 0.00 (−0.07 to 0.10)	0.00 (0.03) 0.00 (−0.08 to 0.10)	0.680	−0.14	−0.81 to 0.53
LogMAR CDVA LE	−0.01 (0.03) 0.00 (−0.08 to 0.00)	0.00 (0.03) 0.00 (−0.07 to 0.09)	0.280	−0.38	−1.05 to 0.31
Phoria at near (Δ)	−8.75 (6.00) −6.00 (−25.00 to –4.00)	−5.90 (1.90) −6.00 (−10.00 to –4.00)	0.070	−0.65	−1.33 to 0.05
NPC break (cm)	12.00 (4.00) 13.00 (6.00 to 18.00)	12.30 (4.70) 12.00 (6.00 to 20.00)	0.410	0.09	−0.64 to 0.82
NPC recovery (cm)	15.70 (5.08) 14.50 (9.00 to 30.00)	13.40 (5.40) 13.00 (7.50 to 25.00)	0.290	0.52	−0.40 to 1.42
NFV at near break (Δ)	13.06 (7.06) 13.00 (4.00 to 35.00)	9.30 (4.60) 9.00 (2.00 to 16.00)	0.070	0.64	−0.06 to 1.32
NFV at near recovery (Δ)	10.80 (5.90) 11.00 (2.00 to 25.00)	7.50 (4.60) 8.00 (1.00 to 14.00)	0.070	0.64	−0.06 to 1.32
PFV at near break (Δ)	15.90 (7.80) 14.00 (6.00 to 30.00)	11.40 (8.20) 12.00 (1.00 to 30.00)	0.110	0.56	−0.13 to 1.24
PFV at near recovery (Δ)	11.90 (5.90) 12.00 (4.00 to 25.00)	7.40 (6.50) 7.00 (0.00 to 25.00)	0.040	0.72	0.02 to 1.41
MAF RE (cpm)	4.40 (4.80) 3.00 (0.00 to 12.00)	4.30 (2.80) 6.00 (0.00 to 8.00)	0.940	0.25	−0.69 to 1.18
MAF LE (cpm)	4.40 (5.00) 3.00 (0.00 to 13.00)	5.00 (3.50) 6.00 (0.00 to 10.00)	0.760	0.11	−0.84 to 1.06
BAF (cpm)	3.30 (3.30) 2.25 (0.00 to 10.00)	2.50 (2.70) 2.00 (0.00 to 8.00)	0.490	0.47	−0.48 to 1.41
Stereopsis (″)	120.00 (65.80) 120.00 (60.00 to 240.00)	82.70 (68.70) 60.00 (30.00 to 280.00)	0.290	0.55	−0.47 to 1.56

SD: standard deviation; RE: right eye; LE: left eye; UDVA: uncorrected distance visual acuity; CDVA: corrected distance visual acuity; NPC: near point of convergence; NFV: negative fusional vergence; PFV: positive fusional vergence; MAF: monocular accommodative facility; BAF: binocular accommodative facility; Δ: prism diopters; cpm: cycles per minute; ″: seconds of arc; CI: confidence interval.

**Table 2:** Summary of visual variables at 30 days of treatment in the two study groups: digital group and conventional group.

Mean (SD) Median (Range)	Digital Group (*n* = 16)	Conventional Group (*n* = 17)	*p*-Value	Cohen’s d	95% CI
Phoria at near (Δ)	−7.30 (3.70) −6.00 (−16.00 to –4.00)	−4.80 (2.00) −5.50 (−8.00 to −2.00)	0.020	−0.89	−1.63 to −0.15
NPC break (cm)	6.30 (5.20) 5.50 (0.00 to 16.00)	1.90 (2.30) 0.00 (0.00 to 5.00)	0.004	1.16	0.29 to 2.01
NPC recovery (cm)	7.30 (5.90) 6.50 (0.00 to 18.00)	1.70 (2.70) 0.00 (0.00 to 6.00)	0.004	1.25	0.26 to 2.22
NFV at near break (Δ)	14.80 (5.50) 14.00 (6.00 to 25.00)	13.10 (3.80) 14.00 (8.00 to 20.00)	0.330	0.36	−0.36 to 1.07
NFV at near recovery (Δ)	11.40 (4.50) 11.00 (4.00 to 20.00)	10.30 (3.30) 10.00 (6.00 to 16.00)	0.440	0.28	−0.43 to 0.99
PFV at near break (Δ)	30.40 (10.20) 32.50 (14.00 to 40.00)	36.50 (5.50) 40.00 (25.00 to 40.00)	0.040	−0.76	−1.49 to −0.02
PFV at near recovery (Δ)	25.90 (12.30) 20.00 (10.00 to 40.00)	32.40 (9.10) 40.00 (16.00 to 40.00)	0.100	−0.61	−1.33 to 0.12
BAF (cpm)	7.00 (3.70) 6.00 (2.50 to 12.00)	9.50 (2.70) 10.00 (4.00 to 13.00)	0.080	−0.44	−1.56 to 0.70

SD: standard deviation; NPC: near point of convergence; NFV: negative fusional vergence; PFV: positive fusional vergence; BAF: binocular accommodative facility; Δ: prism diopters; cpm: cycles per minute; CI: confidence interval.

**Table 3:** Summary of visual variables at 3 months of treatment in the two study groups: digital group and conventional group.

Mean (SD) Median (Range)	Digital Group (*n* = 16)	Conventional Group (*n* = 17)	*p*-Value	Cohen’s d	95% CI
Phoria at near (Δ)	−7.30 (3.70) −6.00 (−16.00 to −4.00)	−4.80 (2.00) −5.50 (−8.00 to −2.00)	0.050	−0.86	−1.71 to 0.01
NPC break (cm)	2.90 (3.03) 2.00 (0.00 to 6.50)	2.20 (2.20) 2.00 (0.00 to 7.00)	0.500	−0.03	−0.94 to 0.88
NPC recovery (cm)	3.40 (3.50) 2.50 (0.00 to 7.50)	2.20 (3.20) 0.00 (0.00 to 9.00)	0.470	0.05	−0.98 to 1.08
NFV at near break (Δ)	15.70 (4.90) 16.00 (10.00 to 25.00)	12.7 (4.000) 12.00 (6.00 to 20.00)	0.110	0.69	−0.17 to 1.54
NFV at near recovery (Δ)	12.90 (4.60) 14.00 (8.00 to 20.00)	9.90 (3.07) 10.00 (4.00 to 16.00)	0.060	0.82	−0.05 to 1.67
PFV at near break (Δ)	35.50 (8.80) 40.00 (20.00 to 40.00)	36.70 (6.50) 40.00 (16.00 to 40.00)	0.710	−0.16	−0.99 to 0.67
PFV at near recovery (Δ)	33.20 (9.80) 40.00 (16.00 to 40.00)	33.60 (7.90) 35.00 (14.00 to 40.00)	0.920	−0.04	−0.87 to 0.78
BAF (cpm)	9.75 (2.70) 10.00 (5.00 to 12.50)	9.40 (1.90) 9.00 (5.50 to 12.00)	0.690	0.00	−0.98 to 0.98

SD: standard deviation; NPC: near point of convergence; NFV: negative fusional vergence; PFV: positive fusional vergence; BAF: binocular accommodative facility; Δ: prism diopters; cpm: cycles per minute.

## Data Availability

Dataset available upon reasonable request from the authors.
